# Informing future seasonal influenza and COVID-19 vaccination campaigns based on past experience: a mixed-method approach with eligible populations and healthcare professionals

**DOI:** 10.3389/fpubh.2025.1616402

**Published:** 2025-08-22

**Authors:** Jorge Oliveira, Bárbara Gonzalez, Teresa Mendes, Paula Paulino, Rui Gaspar, Bruna António, Diana Costa, Leonor Q. Pinto, Andreia Costa, Miguel Telo De Arriaga

**Affiliations:** ^1^Digital Human-Environment Interaction Lab, Lusofona University, Lisbon, Portugal; ^2^Research Center for Psychological Science, Faculty of Psychology, University of Lisbon, Lisbon, Portugal; ^3^Directorate-General of Health (DGS), Lisbon, Portugal; ^4^Nursing Research, Innovation and Development Centre of Lisbon (CIDNUR), Nursing School of Lisbon, Lisbon, Portugal; ^5^Instituto de Saúde Ambiental (ISAMB), Faculdade de Medicina, Universidade de Lisboa, Lisbon, Portugal; ^6^Laboratório para a Sustentabilidade do Uso da Terra e dos Serviços dos Ecossistemas – TERRA, Lisbon, Portugal

**Keywords:** influenza, COVID-19, vaccination intention, seasonal vaccination campaigns, social communication

## Abstract

**Background:**

Seasonal vaccination campaigns against influenza and COVID-19 are critical for protecting vulnerable populations. Scientific evidence on past campaigns is essential for the effectiveness of future campaigns. This study aims to: (1) assess predictors of influenza and COVID-19 vaccination intentions (2) explore perceived barriers and facilitators of 2023–2024 seasonal vaccination campaign.

**Methods:**

A cross-sectional study employing both quantitative and qualitative methods was conducted. The quantitative study involved a sample of 231 respondents from Portugal, including healthcare professionals and vaccination eligible population: individuals over 60 years of age, and individuals with chronic diseases. Quantitative data were collected via an online survey to assess vaccination literacy, attitudes, and future vaccination intention. The qualitative study consisted of four focus groups with a total of 17.

**Participants:**

Vaccinated/unvaccinated individuals and healthcare professionals. Thematic analysis was used to explore perceptions of eligible populations about this vaccination campaign.

**Results:**

Quantitative analysis revealed that attitudes toward vaccination and factors related to personal/family health predicted future vaccination intentions for influenza and COVID-19. Thematic analysis identified key facilitators, such as positive perceptions of pharmacies as vaccination sites and an early start of the campaign specifically for vaccinated groups. Barriers were related to campaign communication, fear of side effects, vaccination fatigue, and the co-occurrence of high temperatures during the campaign start. Unvaccinated individuals expressed lower perceived susceptibility and severity.

**Conclusion:**

The study highlights the importance of clear communication, addressing vaccination fatigue, and considering environmental factors. Improving proximity between health professionals as trusted information sources and the population, alongside customized communication, may also enhance vaccination uptake among the eligible population. The potential influence of weather conditions on the acceptance of vaccination campaigns is a topic that deserves further consideration in the future, within the scope of climate changes.

## Highlights

- The 2023–2024 vaccination campaign against influenza and COVID-19 in Portugal introduced community pharmacies as vaccination centers, which improved accessibility for vulnerable groups.
- Despite high vaccination rates, identified barriers such as high temperatures during the launch of the campaign, unclear information, and fear of side effects contributed to vaccine hesitancy.
- Future influenza vaccination intention among eligible populations was higher than intention for COVID-19.
- Health professionals pointed to a need for tailored communication strategies, suggesting a stronger focus on daytime TV and known presenters to reach older populations effectively.
- Improving vaccination communication strategies and adapting campaign timing in response to weather conditions can enhance vaccine uptake, particularly among hesitant populations.
- The influence of weather events, namely the unexpected high temperatures at the beginning of the vaccination campaign, may have to be addressed in future campaigns.

## Introduction

Vaccination is a decisive pharmacological strategy to prevent infectious diseases and other conditions that put populations at risk (1). The World Health Organization (WHO) recommends the simultaneous vaccination against seasonal influenza and COVID-19, considering their public health benefits (2), and Portugal adopts this strategy since the 2021–22 season.

Considering COVID-19, the fact that it is no longer considered a public health emergency has led to a decreased risk perception among the general population (3), but seasonal vaccinations remain necessary, especially for the most vulnerable population.

Despite the effectiveness of vaccination campaigns (4), concerns and biased beliefs contribute to vaccine hesitancy and refusal, hindering the effectiveness of vaccination programs for the general population (5). The Portuguese population traditionally has shown a low hesitancy toward any vaccine belonging to the National Vaccination Plan (6), and by January 2023 Portugal had the highest COVID-19 vaccination rate in Europe (7). However, in the seasonal 2023–2024 campaign, several European countries had higher rates of COVID-19 vaccination in individuals over 70 years old than Portugal (8), and it has been a decrease in influenza vaccination coverage rates in older adults in Portugal from 2021 to 22 to 2023–24 (9).

The Autumn-Winter 2023–2024 Seasonal Vaccination Campaign against Influenza and COVID-19 in Portugal began in September 2023, featuring several specific changes, including eligibility for free vaccination, vaccination site locations, and a stronger focus on the media communication strategy. It targeted individuals aged 60 years and above (this was updated on January 15 to 50 years), individuals with specific chronic diseases, and specific professional groups including residents, users and professionals from social response facilities, patients and professionals from the integrated continuous care network, National Health System professionals and pregnant women (10).

Therefore, the aforementioned information, and the changes in the 2023–2024 campaign, require an assessment in order to ensure future campaigns will be the most adequate, to maximize vaccination levels.

Informed by the Health Belief Model (11), which integrates risk perception for a disease as a predictor of vaccination intention, and by the Theory of Planned Behavior (12), which states intention as the proxy predictor of behavior, this cross-sectional research aims to: (1) examine a wide set of predictors influencing influenza and COVID-19 vaccination intention in a subset of Portuguese population eligible for free vaccination; (2) provide an in-depth exploration of the perceptions on the 2023–2024 vaccination campaign, identifying perceived vaccination barriers and facilitators. Collecting these two forms of scientific evidence aimed to promote increased effectiveness of future campaigns, by providing information for decision makers of national health authorities.

## Methods

### Study overview

A mixed-method approach was taken, as the best to provide a deeper understanding of complex and dynamic phenomena (13). A quantitative and a qualitative study were conducted simultaneously, grounded, respectively, on an online survey data collection technique and on a focus group data collection technique.

The participants of both studies were selected based on existent vaccination eligibility criteria: (i) individuals over 60 years old; (ii) individuals with chronic diseases covered by DGS standard number 006/2023 (14); or (iii) groups of professionals directly involved in the vaccination process (i.e., doctors, nurses, and pharmacists). Favorable approval to this research was obtained from an Ethics and Deontology Committee for Scientific Research. Data were collected between March and May 2024.

#### Quantitative assessment methods

The sample consisted of a total of 375 respondents fulfilling the eligibility criteria. After removing incomplete cases (i.e., more than 20% of data missing), the final sample consisted of 231 valid cases. Considering the hierarchical linear regression to be performed for identification of potential predictors of future vaccination intention, an *a priori* sample size calculation was conducted on the expected incremental variance (∆R^2^) explained by adding predictors for linear multiple regression. For an anticipated effect size (f^2^) of 0.10 (medium effect size), an alpha level of 0,05, a power of 0,80, for 12 anticipated predictors, the required sample size was 185 participants.

**Figure 1:**
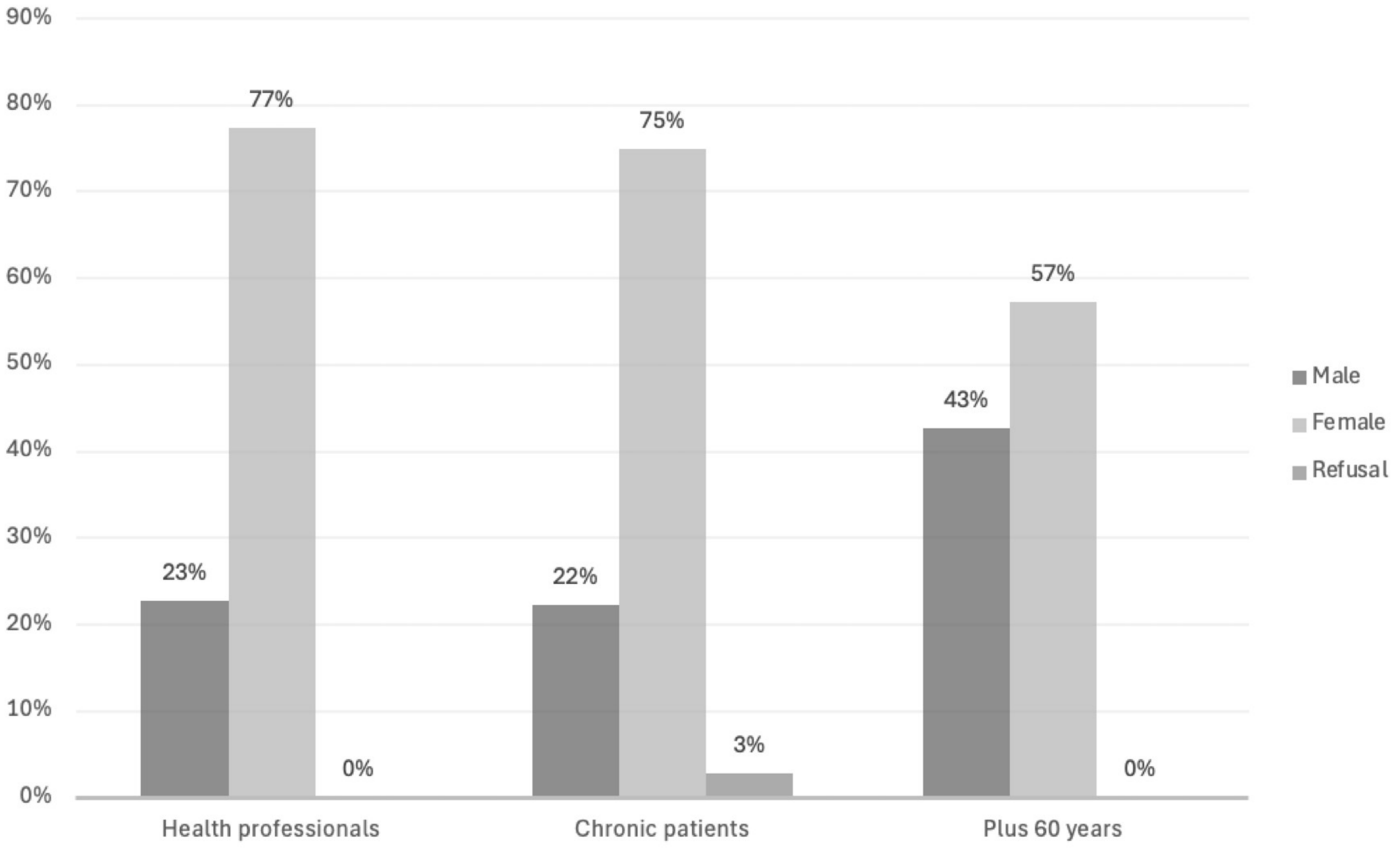
Sex distribution by eligible groups.

**Figure 2:**
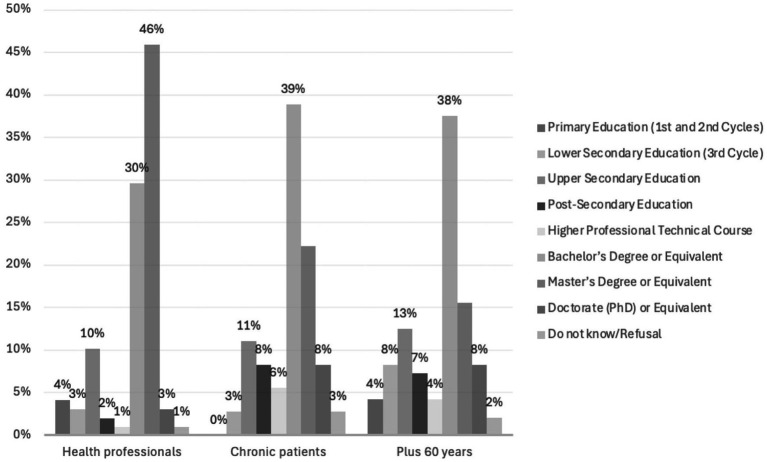
Education distribution by eligible groups.

From the collected sample of 231 participants, 157 (69%) were women and 71 (31%) were men (1 participant did not respond), ranging from 20 to 85 years (*M_age_ =* 52, *SD* = 16.7). The distribution of sex and formal education by eligible groups is shown in Figures 1, 2, respectively.

All analyses were conducted using SPSS (v.28.0). The significance level for all analyses was set at alpha 0.05, corresponding to a 95% confidence level. The statistical analyses began with a correlational analysis using Pearson’s bivariate correlations conducted on the entire sample, examining the relationships between sociodemographic variables, health, contact with health services, the dimensions of the HLS19-VAC measure, and the future influenza and COVID-19 vaccination intention. The variables related to vaccination intention for influenza and COVID-19 were also compared using a paired-samples t-test.

This analysis was followed by two hierarchical linear regression analyses, one for the criterion variable of future influenza vaccination intention, and the other for COVID-19 vaccination intention. Each analysis included all variables with statistically significant correlations, to determine the magnitude of the effect of the predictor variables on the criterion variables. The stepwise method was used for the extraction of predictor variables.

#### Data collection

A questionnaire was developed, comprising three sections: Sociodemographic, Personal health, and Vaccination questions. 1. Sociodemographic [age; sex; school degree; country of birth (own, father, mother)], work (professional status; professional experience in health), socioeconomic (e.g., “Considering Portuguese society, at what socioeconomic level from 1 “lowest level” to 10 “highest level” would you place yourself?”) and social capital (e.g., “How many people do you consider close enough to be able to count on them, if you have serious personal problems?”); 2. Personal health (height; weight; health behaviors), e.g., “In a normal week, how many days do you do X (e.g., smoke or consume tobacco products)—on a scale from “never” to “2 to 7 days”; chronic health conditions; limits to daily tasks due to health problems); contact with health services (e.g., “In the last 12 months, approximately how many times have you sought out a general practitioner, family doctor or health center”). 3. Vaccination: (a) questions framed within the Health Belief Model (perception of severity, susceptibility, benefits, and barriers/facilitators), retrieved from a previous study (15). Examples are: True/False statements—e.g. “Vaccines can cause the disease they are intended to combat”; Likert type scales, as, e.g., “Vaccination is compatible with my religious beliefs” (from 1—Totally disagree to 4—Totally agree), and “How easy or difficult is it to evaluate which vaccines you and your family may need?” (from “Very easy” to “Very difficult.”) (b) A measure to assess vaccination literacy (HLS19-VAC), derived from the HLS19 measure (16); two questions to assess future influenza and COVID-19 vaccination intention: “Do you plan to get a [influenza/COVID] shot next year? Yes–No″.

This questionnaire was administered online through Qualtrics, after a dissemination which prioritized formal contact with social and healthcare structures related to the eligible population.

#### Qualitative assessment methods

Qualitative assessment aimed to provide a deep understanding of how the eligible population to influenza and COVID-19 vaccination perceived the vaccination campaign, to identify barriers and facilitators. The sample included 17 participants who met the study eligibility criteria. The group of individuals over 60 years of age, and with chronic illnesses, consisted of seven participants, the majority of whom were male (*n* = 5; 71.4%) and aged between 52 and 83 years (M = 63.86; SD = 11.23). Four participants received the influenza/COVID-19 vaccination in 2023/24 and three did not. The group of health professionals consisted of 10 participants, mostly female (*n* = 9; 90%) and aged between 25 and 58 years (M = 42.8; SD = 9.70). Six participants received the influenza/COVID-19 vaccination in 2023/24 and four did not. See Appendix A for further information on this sample.

The interviews were transcribed verbatim, resulting in five separate documents: one for each focus group and one combined document for all transcriptions. NVivo® 14 software was used to assist with data management and analysis. The thematic analysis followed the six-phase framework outlined by Braun and Clarke (17). Initially, transcripts were read multiple times to gain familiarity with the data. Relevant features were systematically coded, and these codes were grouped into potential themes. Themes were refined through iterative review, ensuring coherence and alignment with the data. Once defined, themes were supported with representative excerpts in the final report. The process was iterative, allowing for movement between phases to enhance analysis. Codes were developed inductively from participant narratives and deductively from the interview guide and study objectives. Themes and sub-themes were discussed and refined through consensus among two experienced researchers, who independently reviewed the thematic structure to ensure its appropriateness.

### Data collection

Four focus groups were conducted, in order to create homogeneous groups that provide a common ground for discussion while simultaneously allowing for adequate diversity to generate different ideas (18, 19). Both groups (individuals over 60 years old/carriers of chronic diseases, and health professionals) were divided into two, based on the criteria of having received (or not) the influenza/COVID-19 vaccination in 2023/24.

The recruited participants filled out the Informed Consent Form, and the sessions were conducted online via the Zoom platform, with an average duration of 60 min. The sessions were recorded in order to transcribe the responses for later analysis.

The focus group interviews were guided by developed open-ended questions, organized in eight blocks: (a) evolution of risk perception regarding influenza and COVID-19 across time; (b) vaccination scheduling method; (c) pharmacies as new vaccination locations; (d) updated rules on vaccination eligibility; (e) vaccination campaign calendar; (f) impact of weather events on vaccination decision; (g) campaign dissemination means; (h) global perception of the vaccination campaign. This script was the same for patients and professionals, although with the latter responding based on their perception of patients’ attitudes to these issues. The professionals’ script also had a theme related to their perceptions regarding vaccine acceptance.

## Results

### Quantitative assessment results

Bivariate correlations between sociodemographic, socioeconomic, health, contact with health services, vaccination literacy, events impacting vaccination decisions, and future influenza and COVID-19 vaccination intention.

There were statistically significant correlations of future influenza vaccination intention with vaccination knowledge (*r* = 0.280; *p* < 0.001), attitudes toward vaccination (*r* = 0.440; *p* < 0.001), health literacy about vaccination (*r* = 0.201; *p* = 0.007), health-related behaviors such as alcohol consumption (*r* = −0.142; *p* = 0.035), health through days per year in hospitalization (*r* = −0.189; *p* = 0.005), and perceived factors impacting the decision to vaccinate next year related to personal/family health (*r* = 0.359; *p* < 0.001).

Regarding the future intention to vaccinate for COVID-19, statistically significant correlations were found with vaccination knowledge (*r* = 0.260; *p* < 0.001), attitudes toward vaccination (*r* = 0.421; *p* < 0.001), health literacy about vaccination (*r* = 0.180; *p* = 0.017), individual factors such as age (*r* = 0.163; *p* = 0.015), health through days per year in hospitalization (*r* = −0.150; *p* = 0.026), and perceived factors impacting the decision to vaccinate next year related to personal/family health (*r* = 0.391; *p* < 0.001). Table 1 depicts the significant correlations among these variables.

**Table 1:** Bivariate correlations with future vaccination intention for influenza and COVID-19.

	Future vaccination intention for influenza	Future vaccination intention for COVID-19
Attitudes toward vaccination	0.440***	0.421***
Personal/family health impacting the decision to vaccinate next year	0.359***	0.391***
Vaccination knowledge	0.280***	0.260***
Health literacy about vaccination	0.201**	0.180*
Age	0.076	0.163*
Days per year in hospitalization	−0.189**	−0.150*
Alcohol consumption	−0.142*	−0.069

***p-value < 0.001; **p-value < 0.01; *p-value < 0.05.

### Comparison of future influenza and COVID-19 vaccination intentions

A paired-samples t-test was conducted to compare self-reported future intentions for influenza and COVID-19 vaccination among eligible participants in this study. The analysis revealed a statistically significant difference between influenza and COVID-19 vaccination intentions (*t*(220) = 4.269, *p* < 0.001), with higher intention reported for influenza (*M* = 7.75, *SD* = 3.25) than for COVID-19 (*M* = 7.15, *SD* = 3.52). The bivariate correlation between the intentions for influenza and COVID-19 was also significant and strong (*r* = 0.81, *p* < 0.001).

### Linear regression analyses of predictors of future influenza and COVID-19 vaccination intention

In the first analysis, two predictor variables were extracted, explaining *R2a* = 0.232 (23%) of the variance of a statistically significant regression model (*F*(2,164) = 25.813; *p* < 0.001), namely, the variable attitudes toward vaccination and the variable on the factors with the greatest impact on the decision related to personal/family health. Through the standardized coefficients, it was observed that the magnitude of the effect of these predictor variables on the influenza vaccination intention was greater for attitudes toward vaccination directly (*Beta* = 0.390; *t* = 5.607; *p* < 0.001) and then for the factors with the greatest impact on the decision related to personal/family health directly (*Beta* = 0.236; *t* = 3.392; *p* < 0.001).

In the second analysis, three predictor variables were extracted, explaining *R2a* = 0.295 (29%) of the variance of a statistically significant regression model (*F*(3,164) = 23.831; *p* < 0.001), namely, the variable attitudes toward vaccination, the variable age, and the variable on the factors with the greatest impact on the decision related to personal/family health. Through the standardized coefficients, it was observed that the magnitude of the effect of these predictor variables on the COVID-19 vaccination intention was greater for attitudes toward vaccination directly (*Beta* = 0.334; *t* = 4.869; *p* < 0.001), then for the factors with the greatest impact on the decision related to personal/family health directly (*Beta* = 0.311; *t* = 4.541; *p* < 0.001), and age (*Beta* = 0.197; *t* = 2.999; *p* = 0.003). See Table 2 depicting the results from the regression analysis.

**Table 2:** Regression analysis on influenza and COVID future vaccination intentions.

Criterion variables	Adjusted R^2^	Predictors	Standardized beta	*t*
Influenza future vaccination intention	0.232	Vaccination attitudes	0.390	5.607***
		Personal/family health	0.236	3.392***
COVID future vaccination intention	0.295	Vaccination attitudes	0.334	4.869***
		Personal/family health	0.311	4.541***
		Age	0.197	2.999**

Adjusted R2, Coefficient of determination; t, *t*-student test; **p-value < 0.01; ***p-value < 0.001.

### Qualitative assessment results

Findings of the thematic analysis identified four facilitators and seven barriers to vaccination.

#### Facilitators

(1) Susceptibility and severity perception about COVID-19 and/or influenza. Half of the vaccinated > 60 and chronic patients, and health professionals stated that there was a higher susceptibility perception compared with the previous year. Pertaining to severity perception if one gets infected, both these groups consider it is high or very high: “It would be very severe, *(…)* that is why I try at all cost to avoid it,” with professionals stressing it is higher than in the previous year. The unvaccinated > 60 and chronic patients are the only ones with a low perception of susceptibility and severity, half of them stating it is lower than in the previous year.
(2) Enlargement of the vaccination process to pharmacies was indicated by most of the > 60/chronic patients as a positive feature of the 2023–24 campaign. Pharmacies are seen as reliable places by these participants, closer to the eligible population than primary health care centers: “(…) if we can somehow avoid the travels and solve the problem with safety, *it seems that the pharmacy, which is a few steps away from my house, is the best place.*” In the same vein, both vaccinated and unvaccinated professionals perceive this enlargement as a valuable asset to the population: *“(…) pharmacies were a gateway much easier, much less bureaucratic.”* The fact that the population is very accustomed to these facilities was also stressed by the four groups.
(3) Reinforcement of vaccination as a preventive measure to benefit the whole community, associated with the concept of group immunity, was stressed by vaccinated > 60/chronic patients and professionals as the type of message that facilitates vaccination compliance: *“To me, vaccination is a smart and sympathetic choice, not only to ourselves but also to the ones we encounter everyday.”*
(4) Early beginning of the vaccination campaign, before Autumn begins, is considered a positive feature by all groups, except for unvaccinated professionals, which considered the campaign began too early. “*The campaigns must start early, to alert people and to have enough time to sensitize them with enough time*.”

#### Barriers

(1) The high temperature occurring in recent years throughout Autumn was considered by professionals as a relevant motive to keep the population away from the vaccination process: “*It was still too hot, people’s mindset was not ready for this issue yet,”* and also by the unvaccinated > 60/chronic patients: “*There are climate changes that must influence the process…people have their patterns and may be unwilling to accept getting vaccinated in October, if it is like full Summer*.” Therefore, this group suggests the campaign should be reinforced again later.
(2) Lack of clear information about places and schedules for vaccination was presented by the unvaccinated > 60/chronic patients and the two professional groups: *“At first, the start of the process was not very well explained, it was not clear, even for us… it led to much confusion in people.”*
(3) Communication features of the media vaccination campaign were referred by the professional groups and the unvaccinated > 60/chronic patients as not tailored to the target-population, namely a drive toward the social media: “*Social media are a great vehicle, but to young people.”* The professionals also think the campaign should focus on daytime television programs, highly watched by older people, and popular television hosts should be used to communicate the message, instead of the little-known actor in the video of the campaign, considered an ineffective choice: *“(…) they should try that approach, it is so easy to reach people’s homes and use messengers in which they believe. Because sometimes that is what makes people act, “that presenter said that on tv….”* Most of the participants, both professionals and patients, did not recall the advertising video; the few that recalled, did not have a positive opinion, denoting the message was not efficiently transmitted: “the *irony was not very easy to understand, especially among the eligible groups*.” The vaccinated > 60/chronic patients were the only group that did not indicate these negative aspects.
(4) The lack of timely institutional contact to schedule vaccination was stressed, and while almost all of the > 60/chronic patients were contacted, only one of the non-vaccinated patients was. The professionals agree that the lack of contact may be one of the causes for non-vaccination, and being contacted by the primary health care center/pharmacy is potentially more positive than having to be proactive: “*In the last years, that high closeness with the health professionals was a little bit lost, having to search for themselves is different from receiving a message with the right information*.”
(5) Vaccination fatigue was pointed out by both professional groups as a great barrier: *“I believe compliance was hindered by an overload…people said they were sick and tired of vaccines in the last years.”*
(6) Fear of side effects and having some negative experience with vaccines in the past was pointed out by unvaccinated, both > 60/chronic patients and professionals, specifically about COVID-19 vaccine: *“Last year, people had COVID-19 and influenza vaccines at the same time and had a bad reaction. They ended up not knowing which vaccine cause that reaction, and decided not to vaccinate.”*
(7) Having been infected with COVID-19 or influenza at the beginning of the season was perceived as a motive to preclude vaccination, by unvaccinated professionals and one unvaccinated chronic patient. The professionals reinforce that sometimes there is a misperception, as infection happened too long ago to ensure protection, but people still think they may avoid vaccination.

## Discussion

The goals of this study were to: (1) assess predictors of future vaccination intentions for the next seasonal vaccination campaign against influenza and COVID-19 in Portugal; and (2) identify indicators of how vaccination eligible populations and healthcare professionals evaluated the 2023–2024 seasonal vaccination campaign.

Specifically, using a mixed-methods approach, we aimed to obtain an overall perspective of factors influencing vaccination intention within the population at risk as well as among healthcare professionals. Indeed, research has shown that individual’s past vaccination attitudes, intentions and behaviors are associated with present vaccination attitudes, intentions and behaviors (20). Thus, collecting such information, along with perceived barriers and facilitators of vaccine uptake, is key information for health authorities to inform their decisions and define a “course of action” to overcome the former and promote the latter (21).

According to the quantitative findings, attitudes toward vaccination and personal or family health worries were, respectively, the most-important predictors of ensuing influenza and COVID-19 vaccine intentions. This is consistent with previous research based on the Health Belief Model, which states among its constructs that perceived susceptibility and severity strongly impact on health-related behaviors including vaccination (21, 22). Perceived susceptibility and severity are key elements of risk perception, and low perception of risks associated with SARS-CoV-2 infection has been associated with lower vaccination rates [e.g., (23, 24)]. This is coherent with our qualitative finding that the unvaccinated individuals aged > 60 and chronic patients are the only ones with a low perception of susceptibility and severity. This is also aligned with research showing that despite being better protected against illness, the vaccinated persons perceived higher risks than unvaccinated people (25, 26). The latter explain that this may be because unvaccinated persons may really adjust their behavior to minimize risks, they might follow different types of media, or they may minimize the risks exactly to justify their lack of vaccination. As it is evident that risk perception on COVID-19, in general, decreased over time, with the advent of less ill-making variants, and more familiarity with the virus, further exploring unvaccinated persons’ perceptions, and meanings will be increasingly crucial.

Correlations of vaccination knowledge, attitudes and health literacy with vaccination intention, underline the importance of public health communication that effectively conveys the vaccination benefits (27).

The inverse relationship of negative features as alcohol consumption and hospitalization rates, with vaccine intention behaviors, is in accordance with the literature, as those with poorer health-related behaviors or who frequently experience severe health events, seem to be less prone to accepting vaccination. These findings suggest that future campaigns should incorporate tailored strategies to reach these subgroups with potentially lower vaccination intentions (20). Finally, the higher influenza vaccination intention, compared to COVID-19 vaccination intention, is congruent with studies [e.g., (28)] reporting higher influenza vaccination rates in the 2003–24 seasonal campaign, and seems to be related with a general decreased risk perception on COVID-19 since it was no longer considered a public health emergency (3). It contrasts with the results of Santos et al. (5) of a much higher rate of COVID-19 vaccination than influenza vaccination in 2021, when the risk perception on COVID-19 was very high. In the same vein, the fear of side effects of the COVID-19 vaccine as a barrier, emerging in our qualitative results, may be related with greater hesitancy toward this vaccine.

Qualitative findings allowed further exploration on a broad range of facilitators and barriers to vaccination. The fact that the inclusion of community pharmacies as vaccination facilities was so positively evaluated by patients and professionals, perceived as more accessible, more convenient, and less bureaucratic than the formal healthcare settings, seems to indicate the integration of community pharmacies into the vaccination network as a major achievement in the current campaign. This should be continued, and even expanded, in future campaigns, as research has shown that the higher accessibility of a vaccination facility increases the probability that people will get vaccinated (29–31).

Several barriers were identified that may have impacted the vaccination result. A key barrier was related to the perceived ineffectiveness of the media campaign, particularly among healthcare professionals and unvaccinated individuals. As the perception on the campaign quality and the vaccination communication strategies have been considered relevant predictors of vaccination behavior [e.g., (23, 31)], this issue must be improved in future campaigns. The 2023/2024 campaign in Portugal featured a stand-up comedian, who is also a doctor, to deliver the message, and apparently this was not effectively understood by the target population.

The focus on social media, often more used by younger people, may have neglected older adults. Participants suggested that television, particularly programs with well-known presenters, would have been a more effective communication channel for this population stressing the need to align communication strategies with the habits and preferences of the target audience. Our findings highlight the importance of tailoring public health messaging to different demographic groups based on their media consumption habits and trust in information sources. A recent systematic review (32) has found evidence of the beneficial use of emerging technologies in public health communication campaigns, to get a precise segmentation of the audience, increase interactivity with the user in the design of the campaigns, the reach of the message to specific groups, and allow real time adjustments to the contents. Therefore, the use of these technologies could be considered to maximize the social impact of a future vaccination campaign message.

The lack of contact to schedule vaccination, which was a relevant identified barrier, contributes to a lower vaccination convenience perception, which is a relevant predictor of vaccination behavior (31). For future campaigns, this process of setting up appointments could be done using direct outreach methods such as phone or text messages from providers to schedule the vaccine. The issue of vaccination fatigue was an important concern among healthcare professionals. Future campaigns need to consider how to address this fatigue, perhaps through more personalized vaccination schedules, or by emphasizing the long-term benefits of vaccination over the cumulative burden.

Finally, the timing of the campaign, which began early in autumn, was perceived by some participants, specifically the group of unvaccinated professionals, as too early due to unexpected warm temperatures, which highlights the potential influence of weather events such as extreme temperatures on public health campaigns. To our best knowledge, no study has yet addressed this issue, which may tend to be increasingly relevant within the scope of climate changes. Future campaigns may need to be more flexible and responsive to weather conditions to better align public health messaging with the population’s vaccination intention.

Our results suggest that unvaccinated individuals with chronic diseases, despite at higher risk of poor health outcomes, have a lower susceptibility and severity perception about influenza and COVID-19. This is in line with research showing that chronic patients tend to exhibit lower vaccine uptake intentions [e.g., (33, 34)]. Future studies should explore the psychological factors influencing vaccine decision-making in medically vulnerable population, to better inform targeted strategies and education about COVID-19/Influenza vaccination to support these populations. As the fear of side effects, a relevant predictor of non-vaccination [e.g., (23, 29)], is also a barrier identified by unvaccinated individuals in our study, accurate information on possible side effects and their consequences may decrease this barrier.

There were some limitations related to the sample sizes in both studies, which may have impacted the generalizability of the findings. The online format in both studies, although enabling more representativity of participants from different geographical areas, may have limited adherence, considering the age range of the preferred target group of the campaign. The low number of participants in the qualitative study is relevant, and older individuals may have retreat from participating due no only to difficulties with online literacy and even to discomfort with the online format.

We accessed vaccination intention, and it must be present that high willingness to receive the vaccination may correspond to lower levels of actual vaccination behavior (29).

Despite these limitations, the study provides valuable insights about factors affecting vaccination uptake and proposes recommendations for upcoming vaccination campaigns.

In sum, this study highlights the multifaceted nature of vaccination hesitancy (5, 20). While the quantitative data provided a reflection into predictors of future vaccination intention, the qualitative data enhanced our understanding of the specific barriers/facilitators faced by individuals. Accounting for these two factors together may empower future vaccination campaigns in Portugal and in other countries, to target specific subgroups. Given that vaccine facilitators might be context-specific and vary significantly between populations, regions or cultures, understanding these factors allows the development of more effective, culturally sensitive public health campaigns, ultimately contributing to healthier societies throughout the world.

## Data Availability

The raw data supporting the conclusions of this article will be made available by the authors, without undue reservation.
