# Supplementary material for: Informing future seasonal influenza and COVID-19 vaccination campaigns based on past experience: a mixed-method approach with eligible populations and healthcare professionals
Source: Front Public Health. 2025 Aug 22;13:1616402. doi: 10.3389/fpubh.2025.1616402 (PMC12411470; doi:10.3389/fpubh.2025.1616402)
Supplement: Supplementary file 1 [file Table_1.docx]

Appendix A. Sociodemographic and health characterization of focus groups participants

Individuals over 60 years of age/with chronic diseases

|  | Age | Gender | Marital Status | Educational Level | Profession | Nr. of consultations in the NHS/year | Chronic illness | Time since diagnosis | Degree to which the illness(es) affect life | Vaccinated for COVID/ influenza in 2023/24 |
| --- | --- | --- | --- | --- | --- | --- | --- | --- | --- | --- |
| P1 | 68 | Male | Divorced | Middle School | Retired | 10-20 | Yes (COPD, hypertension) | 6 years; 20 years | A lot | Yes |
| P2 | 73 | Male | Married | High School | Retired | 1-5 | Yes (COPD) | 23 years | Little | Yes |
| P3 | 64 | Male | Married | Bachelor’s | Retired | 5-10 | Yes (COPD) | 7 years | A lot | Yes |
| P4 | 52 | Female | Married | Doctorate | Consultant | 10-20 | Yes (rheumatoid arthritis, hypertension) | 47 years | A lot | Yes |
| P5 | 83 | Male | Widower | High School | Retired | 1-5 | No | - | - | No |
| P6 | 59 | Male | Married | Master’s | Retired | 5-10 | Yes (multiple sclerosis) | More than 30 years | A lot | No |
| P7 | 48 | Female | Single | Bachelor’s | Jurist | 1-5 | Yes (systemic lupus erythematosus) | 8 years | Little | No |

*Note*. NHS = National Health System; COPD = Chronic obstructive pulmonary disease

Health professionals

|  | Age | Gender | Marital Status | Educational Level | Profession | Time in profession | Perception of health status | Chronic illness | Vaccinated for COVID/ influenza in 2023/24 |
| --- | --- | --- | --- | --- | --- | --- | --- | --- | --- |
| P1 | 42 | Female | Married | Master’s | Nurse | 20 years | Great | No | Yes |
| P2 | 52 | Female | Married | Bachelor’s | Pharmacist | 26 years | Great | No | Yes |
| P3 | 37 | Female | Married | Master’s | Doctor | 11 years | Great | No | Yes |
| P4 | 41 | Female | Married | Bachelor’s | Pharmacist | 18 years | Very good | No | Yes |
| P5 | 33 | Female | Single | Master’s | Doctor | 5 years | Good | No | Yes |
| P6 | 25 | Female | Single | Bachelor’s | Nurse | 4 years | Great | No | Yes |
| P7 | 42 | Male | Married | Bachelor’s | Pharmacist | 17 years | Great | No | No |
| P8 | 56 | Female | Divorced | Master’s | Pharmacist | 30 years | Very good | No | No |
| P9 | 58 | Female | Married | Master’s | Pharmacist | 34 years | Good | No | No |
| P10 | 42 | Female | Married | Master’s | Nurse | 13 years | Very good | No | No |

*Note*. NHS = National Health System
